# Regulation of Interferon-β by MAGI-1 and Its Interaction with Influenza A Virus NS1 Protein with ESEV PBM

**DOI:** 10.1371/journal.pone.0041251

**Published:** 2012-07-20

**Authors:** Manish Kumar, Hongbing Liu, Andrew P. Rice

**Affiliations:** Department of Molecular Virology and Microbiology, Baylor College of Medicine, Houston, Texas, United States of America; University of Ottawa, Canada

## Abstract

The NS1 protein from avian influenza A viruses contains a PDZ binding motif (PBM) at its carboxyl terminus with the consensus sequence ESEV. The ESEV PBM confers binding to several cellular PDZ proteins, including Dlg1, MAGI-1 and Scribble. The interaction between NS1 and Scribble protects infected cells from apoptosis, while the interaction between NS1 and both Dlg1 and Scribble disrupts tight junctions. In this study, we examined the MAGI-1 protein. We made the unexpected observation that siRNA depletion of MAGI-1 activates IRF3 and induces the IFN-β promoter. We found that the ESEV NS1 protein sequesters MAGI-1 away from the plasma membrane in infected cells. Using plasmid vectors to express NS1 proteins, we observed that the ESEV PBM elicits an IFN-β induction signal as indicated by activation of IRF3 and a relative deficiency in NS1 inhibition of induction of the IFN-β promoter by dsRNA or RIG-I. Taken together, our data suggest that disruption of MAGI-1 by the ESEV PBM activates an IFN-β induction signal. During viral infection, however, induction of the IFN-β gene does not occur presumably because other anti-IFN functions dominate over the IFN-activation activity of the ESEV PBM. We postulate that the ESEV PBM's broad binding activity for PDZ proteins may allow NS1 to bind to some PDZ proteins such as MAGI-1 that confer no benefit or may even be detrimental to viral replication. However, the advantage of binding to key PDZ proteins such as Dlg1 and Scribble may dominate and therefore provide an overall benefit for the virus to encode the ESEV PBM.

## Introduction

The PDZ domain is a protein-protein interaction module found throughout evolution, from bacteria to metazoans [1]. PDZ proteins often contain multiple PDZ domains and additional protein interaction domains such as SH3, L27, or WW domains. PDZ proteins are typically found in the cytoplasm or associated with the plasma membrane and are involved in a variety of cellular processes of significance to viruses, such as cell-cell junctions, cellular polarity, and signal transduction pathways. PDZ domains usually bind to a specific four amino acid residue sequence at the carboxyl terminus of a target protein, termed the PDZ binding-motif (PBM) in the target protein. It has become appreciated in recent years that viruses from many viral families encode proteins with PBMs, indicating that viruses commonly target cellular PDZ proteins to enhance their replication, dissemination in the infected host, or transmission to new hosts [2]. These interactions between viral proteins and their PDZ targets often result in the degradation or sequestration of the cellular protein. For example, the human papillomavirus-16 E6 protein binds to the PDZ proteins Scribble and Dlg1 and this leads to proteasome-mediated proteolysis of the cellular proteins [3], [4]. The adenovirus E4 Orf1 protein binds to the PDZ protein MUPP1 and the human T cell leukemia virus Tax proteins bind to Dlg1, and in both cases the viral protein sequesters the cellular PDZ protein in aberrant detergent-insoluble structures.

A large-scale sequencing study identified a PBM at the carboxyl terminus of the influenza A virus NS1 protein [5]. In avian viral isolates, the consensus NS1 PBM sequence is ESEV (∼80% of viral isolates), while that of human viral isolates is RSKV (∼85% of viral isolates). The current circulating highly pathogenic H5N1 influenza A viruses generally encode an NS1 protein with the ESEV PBM sequence. In contrast, the recent 2009 swine-origin H1N1 pandemic virus encodes an NS1 with a deletion of the PBM. Both the ESEV and RSKV PBMs can function as virulence determinants in infected mice [6], although there are cell-type and species-specificity effects on the PBM's contribution to virulence [7].

A number of PDZ protein targets of the NS1 ESEV PBM have been identified though a variety of protein-binding assays: Dlg1, MAGI-1, MAGI-2, MAGI-3, Scribble, Lin7C, PDLIM2, PSD-95 [8]–[12]. The interaction between NS1 and Scribble inhibits Scribble's pro-apoptotic function and thereby protects infected cells from apoptosis [12]. The interaction between NS1 and both Scribble and Dlg1 disrupts cellular tight junctions during infection and this likely contributes to viral pathogenesis [11]. Both Scribble and Dlg1 are sequestered with NS1 in perinuclear cytoplasmic puncta that partition into the insoluble cell fraction upon detergent lysis [12]. The functional significance of the interaction between NS1 and the other PDZ targets remains to be established, and PDZ targets of the RSKV PBM remain to be identified.

As well as being capable of encoding a PBM, the influenza A virus NS1 protein associates with a number of cellular proteins that have antiviral functions and this inhibits the host innate immune response to infection [13], [14]. A key step in the innate immune response to viral infection is the activation of the type I Interferon (IFN) pathway and induction of IFN-Stimulated Genes (ISGs) that establish the antiviral state [15]. Activation of the IFN pathway can be triggered by Toll-Like Receptors (TLRs), Nod-Like Receptors (NLRs), or RNA helicases such as RIG-I and MDA-5 [16], [17]. The RNA helicases are activated by dsRNA structures produced during the replication of RNA viruses. In the case of influenza A virus, RIG-I has been shown to be a major sensor for viral dsRNA [18]. Activated RIG-I interacts with the MAVS protein (also known as IPS-1, Cardiff, and VISA) [19]–[22], and this leads to the activation of IFN regulatory factors IRF3/7 and NF-κB, which then induce expression of IFN-α and IFN-β genes. Additionally, the TRIM25 protein has been shown to ubiquitylate RIG-I and enhance induction of IFN [23].

The influenza A virus NS1 protein consists of 230–237 amino acid residues and contains two structural domains, the amino terminal RNA Binding Domain and the carboxyl terminal Effector Domain [24]–[27]. NS1 can suppress induction of IFN by two distinct pathways. NS1 binds directly to Cleavage and Polyadenylation Specificity Factor 30 (CPSF30) and inhibits maturation of IFN and other cellular mRNAs in the nucleus [28]. NS1 also binds to TRIM25 and inhibits ubiquitylation of RIG-I and this reduces the induction of IFN mRNA [29]. Similar to many viruses, influenza A virus encodes more than a single protein with an IFN antagonist function. PB2, a subunit of the influenza virus RNA polymerase, interacts with MAVS and inhibits IFN-β expression [30]. The viral PB1-F2 inhibits RIG-I-mediated induction of IFN by suppressing MAVS function [31].

In the present study, we investigated the interaction between NS1 with the ESEV PBM and the PDZ protein known as MAGI-1. We made the unexpected observation that siRNA depletion of MAGI-1 activates IRF3 and induces the IFN-β promoter. We also found that the ESEV NS1 protein sequesters MAGI-1 in perinuclear puncta in infected cells. Using a number of assays to quantify induction of IFN-β, we unexpectedly found that when expressed from a plasmid vector, the ESEV PBM not only reduces the ability of NS1 to inhibit IFN-β but also elicits a signal for IFN-β induction. In the context of viral infection, however, other anti-IFN viral functions such as the inactivation of CPSF30 by NS1 masks this ESEV PBM function so that IFN-β induction is blocked. Our data suggests that inactivation of MAGI-1 by NS1 elicits an IFN-β induction signal, but other viral anti-IFN functions dominate over this activity to suppress IFN-β induction. Our results identify for the first time that the PDZ protein MAGI-1 is a regulator of IFN-β induction and highlight the benefit and perhaps requirement for influenza A virus to encode multiple anti-IFN functions and proteins.

## Results

### SiRNA depletion of MAGI-1 activates the IFN-β promoter and IRF3

**Figure 1:**
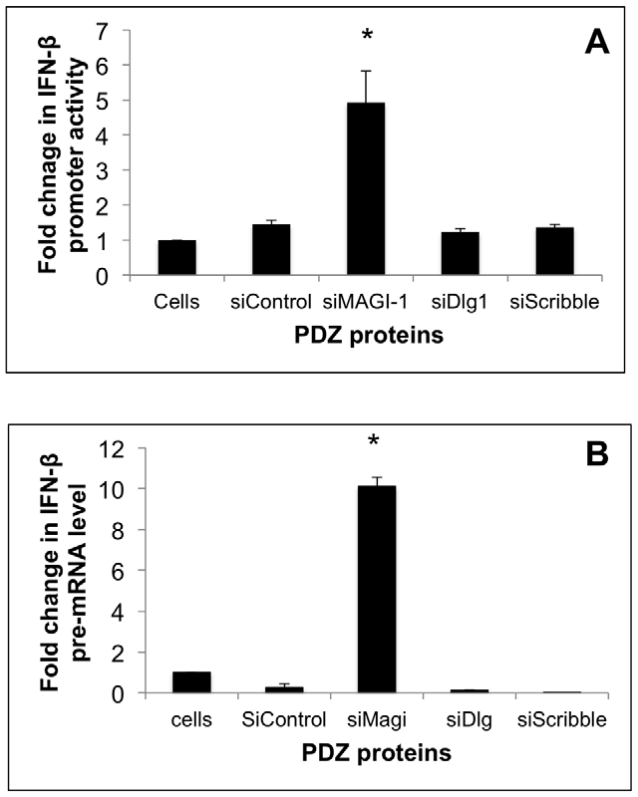
Depletion of MAGI-1 activates IFN-β promoter. A) A549 cells were transfected in duplicate with siRNAs against MAGI-1, Dlg-1, Scribble, and control siRNAs; lanes labeled “Cells” are non-transfected control cells. Cells were re-transfected 24 hours later with IFN-β Luciferase and Renilla Luciferase plasmids and Luciferase expression was measured 24 hours later. IFN-β Luciferase expression was normalized to Renilla Luciferase. Error bars represent the standard error of the mean from three independent experiments, with each experiment containing duplicate samples. Statistical difference in effects of NS1 plasmids was determined by student *t-test* in all the experiments. B) A549 cells were transfected in duplicate with the indicated siRNAs. At 24 hours post-transfection, total RNA was isolated and used in RT-PCR assays to quantify IFN-β pre-mRNA levels. IFN-β pre-mRNA levels were normalized to GAPDH mRNA levels. Error bars represent the standard error of the mean from three independent experiments, with each experiment containing duplicate samples.

We were interested in examining if cellular PDZ proteins targeted by the influenza A NS1 ESEV PBM might have functional roles in IFN induction, as the interaction between the TBEV NS5 protein and Scribble interferes with IFN-stimulated JAK-STAT signaling [32]. We therefore used siRNAs to deplete select PDZ protein targets in A549 cells and measured the effect on a transfected IFN-β promoter Luciferase plasmid. We depleted MAGI-1, Dlg1, and Scribble – three proteins that we have previously reported to bind to NS1 with the ESEV PBM in GST pull-down assays [12]. Depletions of Dlg1 and Scribble did not activate the IFN-β reporter plasmid (Fig. 1A). In contrast, depletion of MAGI-1 resulted in an approximate 5-fold induction of the IFN-β Luciferase reporter. As an independent assay for activation of IFN-β, we use a RT-PCR assay to quantify IFN-β pre-mRNA levels following depletion of MAGI-I, Dlg1, and Scribble (Fig. 1B). In agreement with activation of the IFN-β promoter Luciferase plasmid, depletion of Dlg1 and Scribble had no effect on IFN-β pre-mRNA levels, while depletion of MAGI-1 increased the level approximately 10-fold.

**Figure 2:**
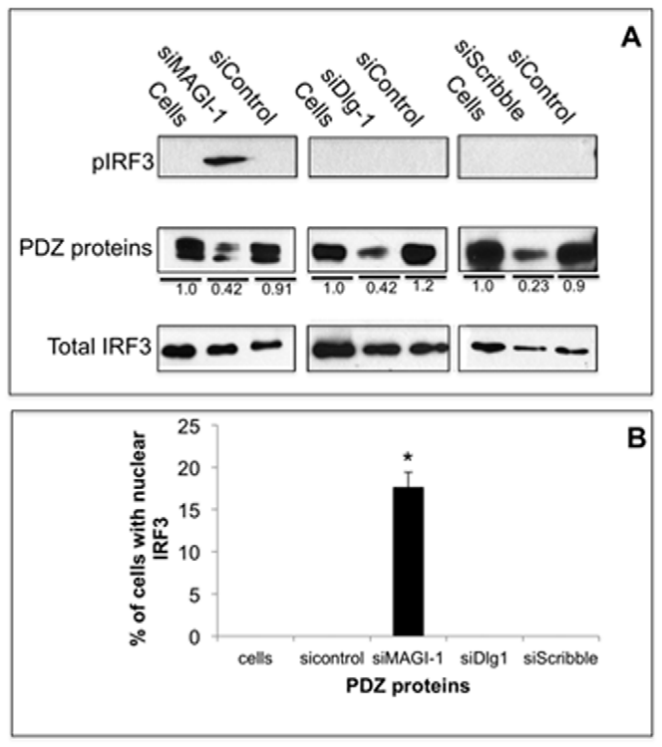
Depletion of MAGI-1 activates IRF3. A) A549 cells were transfected with the indicated siRNAs against MAGI-1, Dlg-1, Scribble, and control siRNAs; lanes labeled “Cells” are non-transfected control cells. After 48 hours, cells extracted were prepared and the levels of indicated proteins were examined in immunoblots. The levels of proteins were measured by densitometry analysis using ImageJ software; the signal for each PDZ protein in non-transfected control cells was arbitrarily assigned a value of 1.0 and PDZ protein levels in transfected cells is shown relative to this 1.0 value. B) A549 cells were transfected with the indicated siRNAs. After 24 hours, cells were processed for immunofluorescence for total IRF3 as described in Methods for three independent experiments. Percentages of cells with nuclear localized-IRF3 were quantified by manually counting of at least 200 cells (a representative image for this assay is shown in Figure 7C). Error bars represent the standard error of the mean from three independent experiments, with each experiment containing duplicate samples.

We next examine the effect of siRNA depletions of MAGI-1, Dlg1, and Scribble in A549 cells on phosphorylation and nuclear translocation of IRF3. Activation of IFN-β involves phosphorylation of IRF3 by the IKK-like kinases TBK-1 and IKKε [33]. Phosphorylated IRF3 (pIRF3) then dimerizes and translocates to the nucleus where it acts to stimulate RNA polymerase II transcription of IFN-regulated genes [33]. Although there were significant decreases in these PDZ proteins by their respective siRNA, only depletion of MAGI-1 induced pIRF3 levels as examined in immunoblots (Fig. 2A). We also used immunofluorescence to quantify nuclear translocation of IRF3. In agreement with the immunoblot to measure pIRF3, the depletion of MAGI-1 but not the other PDZ proteins resulted in nuclear translocation of IRF3 in approximately 18% of cells (Fig. 2B). We conclude from these data that depletion of MAGI-1 results in activation of IRF3 and the IFN-β promoter. To our knowledge, this is the first observation that MAGI-1 is involved in the IFN pathway.

### NS1 with ESEV PBM associates with MAGI-1 and NS1 co-localizes with MAGI-1 and Scribble in perinuclear puncta in infected cells

**Figure 3:**
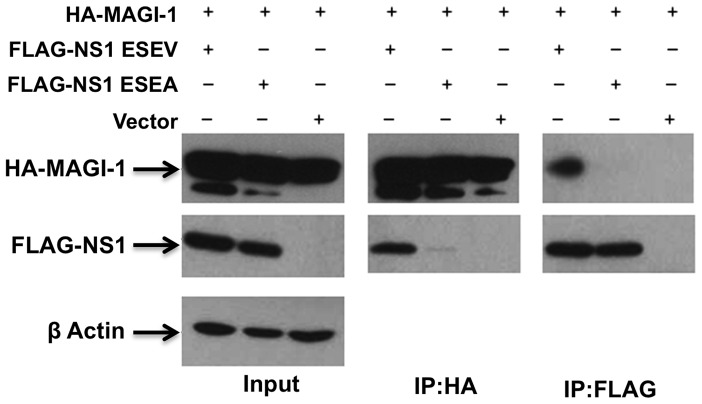
ESEV PBM confers interaction between NS1 and MAGI-1. 293T cells were transfected with HA-MAGI-1 and either FLAG-H6N6 NS1 ESEA or FLAG-H6N6 NS1 ESEV plasmids. At 48 hours post-transfection cell extracts were prepared and used in immunoprecipitations with anti-HA or anti-FLAG antibody. Products of immunoprecipitations were examined in immunoblots.

We previously reported that the H6N6 NS1 protein with the wt ESEV PBM but not mutant ESEA PBM associates with MAGI-1 in GST pull-down assays [12]. To confirm that this association occurs in cells, 293T cells were transfected with FLAG tagged wt and mutant NS1 and HA-MAGI-1 plasmids. After immunoprecipitation with an HA antibody for MAGI-1, the wt NS1 but not ESEA NS1 were found to co-immunoprecipitate in an immunoblot (Fig. 3). In a reciprocal experiment with a FLAG antibody to immunoprecipitate NS1, MAGI-1 was found to co-immunoprecipitate with the wt but not mutant NS1 (Fig. 3). The results of these co-immunoprecipitation experiments confirm that the interaction between NS1 and MAGI-1 in cells requires the ESEV PBM.

**Figure 4:**
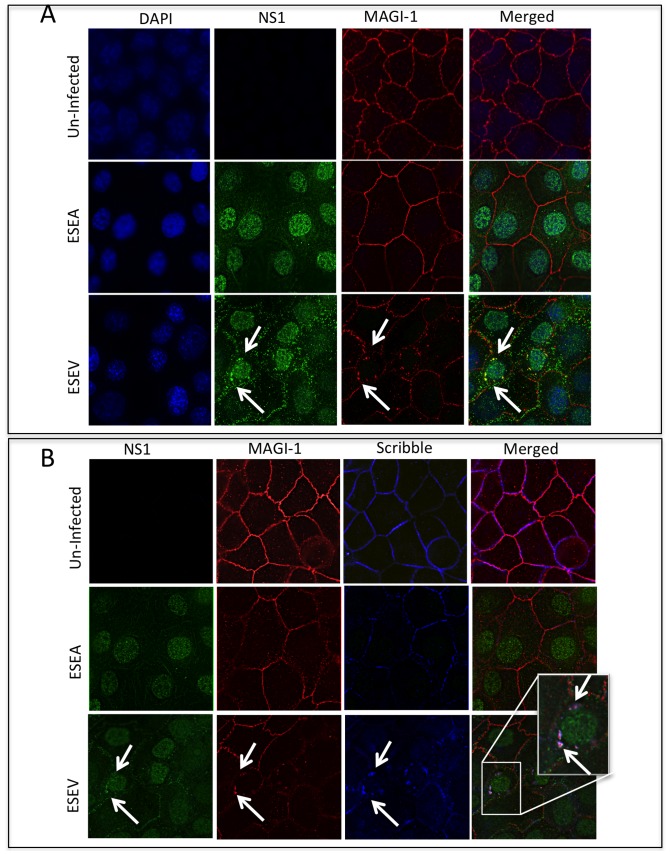
NS1 with ESEV PBM co-localizes with MAGI-I and Scribble in cytoplasmic puncta. A) A549 cells were infected with wt or ESEA mutant virus at an m.o.i. of 1. Cells were processed for immunofluorescence at 24 hours post-infection. Arrows indicate perinuclear puncta where ESEV NS1 and MAGI-1 co-localize. B) Same image as panel A; NS1 is shown as green, MAGI-1 is shown as red, and Scribble is shown as red. Arrows indicate puncta containing co-localization of NS1, MAGI-1, and Scribble.

We also used immunofluorescence to evaluate the association of NS1 with MAGI-1 during influenza virus infection. For this experiment, we used recombinant H3N2 influenza A viruses (Udorn strain) encoding an H6N6 NS1 protein with either the wt ESEV PBM or mutant ESEA PBM [11], [12]. A549 cells were infected with either the wt NS1 or mutant ESEA virus and localization of NS1 and MAGI-1 was examined at 24 hours post-infection. MAGI-1 was localized at the plasma membrane in mock-infected and ESEA virus-infected cells (Fig. 4A). In contrast, the plasma membrane localization of MAGI-1 was perturbed in wt NS1-infected cells and co-localization of NS1 and MAGI-1 could be observed in perinuclear puncta. We previously observed co-localization of NS1 with Dlg1 and Scribble in perinuclear puncta in wt infected cells but not mutant ESEA-infected cells [11]. We also observed in this study co-localization of MAGI-I and Scribble in perinuclear puncta in wt but not mutant NS1-infected cells (Fig. 4B). Figure 4A and 4B present the same immunofluorescence image; NS1 (rabbit antiserum) and MAGI-1 (mouse antiserum) are shown as green and red, respectively, in both panels, while Scribble (goat antiserum) is shown as blue in panel B.

### NS1 inhibition of IFN-β promoter induction by poly(I:C) or RIG-I is antagonized by the ESEV PBM

The results presented in Figure 1 above indicate that siRNA depletion of MAGI-1 results in an IFN-β induction signal. The immunofluorescence images shown in Figure 4 indicate that the association between this PDZ protein and NS1 causes the sequestration of MAGI-1, perhaps resulting in the functional inactivation of MAGI-1. The consequence of this putative functional inactivation may be similar to the siRNA depletion of MAGI-1 – that is, the generation of an IFN-β induction signal. We therefore wished to examine whether the ESEV PBM affected the ability of NS1 to antagonize activation of the IFN-β promoter by dsRNA or RIG-I. To enhance expression levels from H6N6 NS1 plasmid vectors and allow stable expression of an internal control Renilla Luciferase protein in this experiment, we introduced a mutation that abolishes the CPSF30 binding site in the NS1 Effector Domain, as binding of NS1 to CPSF30 inhibits 3′ end processing of pre-mRNA from plasmid vectors [34], [35].

**Figure 5:**
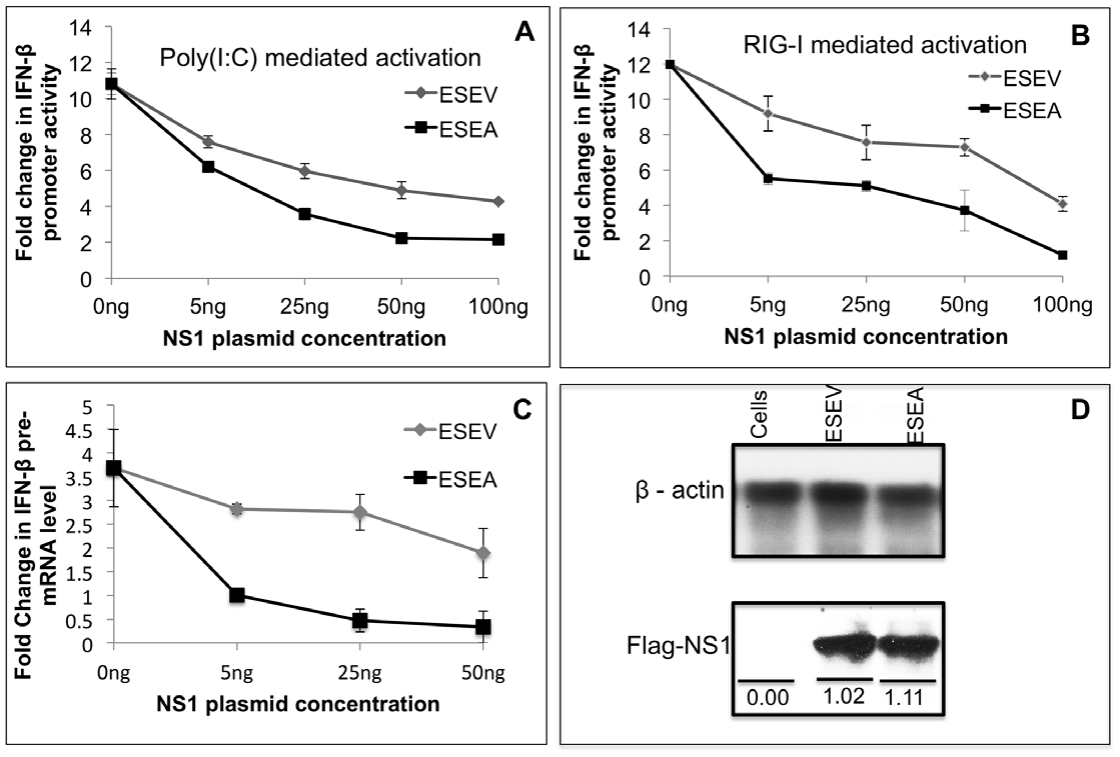
ESEV PBM impairs NS1 inhibition of IFN-β promoter activation. A) Cultures of A549 cells were transfected in duplicate with indicated amounts of NS1 expression plasmids (containing inactivated CPSF30 binding site), IFN-β promoter Luciferase plasmid, and Renilla Luciferase plasmid. At 24 hours post-transfection, cells were re-transfected with poly(I:C) and Luciferase expression was measured 20 hours later. Luciferase expression from the IFN-β promoter plasmid was normalized to Renilla Luciferase expression. Error bars represent the standard error of the mean from three independent experiments, with each experiment containing duplicate samples. B) Cultures of A549 cells were co-transfected in duplicate with indicated amounts of NS1 expression plasmids (containing inactivated CPSF30 binding site), IFN-β promoter Luciferase plasmid, Renilla Luciferase plasmids and, full-length RIG-I expression plasmid. Luciferase expression from the IFN-β promoter plasmid was normalized to Renilla Luciferase expression. Error bars represent the standard error of the mean from three independent experiments, with each experiment containing duplicate samples. Statistical differences in effects of NS1 plasmids were determined by student *t-test* in all the experiments. C) Cultures of A549 cells were transfected in duplicate with indicated amounts of NS1 expression plasmids (containing intact CPSF30 binding site). At 24 hours post-transfection, cells were re-transfected with poly(I:C) and 24 hours later total RNA isolated and RT-PCR assays were performed for IFN-β pre-mRNA as described in Methods. IFN-β pre-mRNA levels were normalized to GAPDH mRNA levels. Error bars represent the standard error of the mean from three independent experiments, with each experiment containing duplicate samples. D) A549 cells were transfected with 500 ng of wt or ESEA mutant NS1 expression plasmids (containing inactivated CPSF30 binding site). Cell extracts were prepared 48 hours later and expression levels of the Flag-tagged NS1 proteins were evaluated in an immunoblot. Densitometry analysis was performed by using ImageJ software. Values shown were normalized to corresponding internal control β-actin protein.

To evaluate the effect of the NS1 PBM on induction of the IFN-β promoter by dsRNA, we transfected A549 cells with increasing amounts of NS1 expression plasmids along with an IFN-β promoter Luciferase plasmid and an internal control Renilla Luciferase expression plasmid. At 24 hours post-transfection, cells were re-transfected with synthetic double-strand RNA, poly(I:C), and Luciferase expression was measured 20 hours later (Fig. 5A). In this experiment, poly(I:C) stimulated the IFN-β promoter plasmid approximately 11-fold in the absence of an NS1 expression plasmid. Although both the wt and mutant ESEA NS1 proteins inhibited induction of the IFN-β promoter in a dose-dependent manner, the PBM mutant was unexpectedly significantly (p<0.01) more active for inhibition.

To evaluate RIG-I activation of the IFN-β promoter, A549 cells were transfected with increasing amounts of the wt or mutant NS1 expression plasmid (with mutant CPSF30 binding site), a full-length RIG-I expression plasmid, the IFN-β Luciferase plasmid, and Renilla Luciferase internal control. Luciferase expression was measured at 24 hours post-transfection (Fig. 5B). In this experiment, RIG-I activated the IFN-β Luciferase plasmid approximately 12-fold in the absence of an NS1 expression plasmid. Similar to induction of the IFN-β promoter by dsRNA, the mutant ESEA NS1 protein was significantly (P<0.01) more active than the wt NS1 in inhibition of RIG-I activation. The data presented in Figure 5A and 5B suggest that the ESEV PBM in the H6N6 NS1 protein antagonizes the ability of NS1 to inhibit activation of the IFN-β promoter by dsRNA or RIG-I.

**Figure 6:**
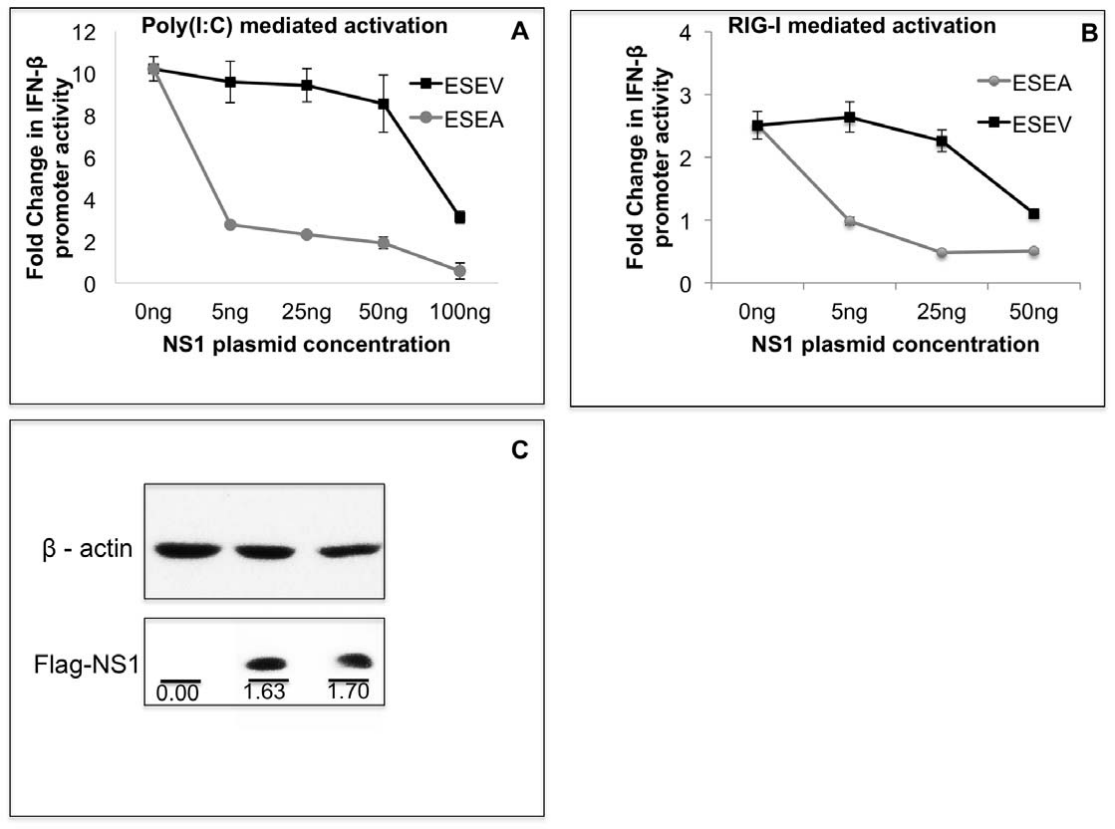
ESEV PBM with intact CPSF30 binding site impairs NS1 inhibition of IFN-β promoter activation. A) Cultures of A549 cells were transfected in duplicate with indicated amounts of NS1 expression plasmids (containing intact CPSF30 binding site), IFN-β promoter Luciferase plasmid, and Renilla Luciferase plasmid. At 24 hours post-transfection, cells were re-transfected with poly(I:C) and Luciferase expression was measured 20 hours later. Luciferase expression from the IFN-β promoter plasmid was normalized to protein concentration. Error bars represent the standard error of the mean. B) Cultures of A549 cells were co-transfected in duplicate with indicated amounts of NS1 expression plasmids, IFN-β promoter Luciferase plasmid, Renilla Luciferase plasmids and, full-length RIG-I expression plasmid. Luciferase expression from the IFN-β promoter plasmid was normalized to protein concentration. Error bars represent the standard error of the mean. Statistical difference in effects of NS1 plasmids was determined by student t-test in all the experiments. C) A549 cells were transfected with wt and mutant ESEA NS1 plasmid with intact CPSF30 site. After 48 hours, cells were lysed and analyzed for expression of NS1 in an immunoblots. The NS1 protein levels were normalized to corresponding β-actin level. Densitometry analysis was performed by ImageJ software.

We also examined plasmid vectors that express the wt and mutant ESEA NS1 proteins with an intact CPSF30 binding site (Fig. 6). In transfections with plasmids that express NS1 proteins with an intact CPSF30 binding site, Luciferase expression from the IFN-β promoter plasmid was normalized to protein concentration of cell lysates because these NS1 proteins repress the internal control Renilla Luciferase expression through inhibition of 3′ end RNA processing of the plasmid vector. Identical to the results shown in Figure 5, activation of IFN-β promoter by Poly(I:C) or RIG-I was significantly more inhibited by the mutant ESEA NS1 protein than the wt ESEV protein (Fig. 6A,6B). We also examined the effect of the ESEV PBM on induction of the IFN-β promoter in 293T cells and found that activation of the IFN-β promoter by poly(I:C) or RIG-I was more potently inhibited by the mutant ESEA NS1 protein than the wt PBM protein, irrespective of a mutant or intact CPSF30 binding site (data not shown). Taken together, these data indicate that the ESEV PBM reduces the ability of the H6N6 NS1 protein to inhibit activation of the IFN-β promoter, and this property is independent of NS1 binding to CPSF30.

### NS1 inhibition of induction IFN-β pre-mRNA levels by poly(I:C) is antagonized by the ESEV PBM

We used an RT-PCR assay developed by the Krug laboratory to examine the effect of the ESEV PBM on IFN-β pre-mRNA levels [36]. In this experiment, we used NS1 plasmid vectors that express NS1 proteins that bind to CPSF30 and inhibit 3′ end RNA processing. When NS1 binds and sequesters CPSF30, very little IFN-β pre-mRNA is processed to the mature form of IFN-β mRNA. Importantly, measurements of IFN-β pre-mRNA levels quantify the effect of the NS1 PBM on activation of transcription of the IFN-β gene and not production of mature IFN-β mRNA. A549 cells were transfected with wt or ESEA NS1 plasmids followed by re-transfection with poly(I:C) 24 hours later, and 24 hours later total cellular RNA was isolated for the RT-PCR assay to quantify IFN-β pre-mRNA. Consistent with results with the IFN-β Luciferase reporter plasmid in Figure 5A and 5B, the mutant ESEA NS1 protein demonstrated a greater inhibition in IFN-β pre-mRNA level relative to the wt NS1 protein (Fig. 5C). An immunoblot analysis demonstrated that the wt and ESEA mutant NS1 proteins were expressed at similar levels in A549 cells (Fig. 5D). Taken together, the data shown in Figure 5 indicate that when expressed from a plasmid vector, the ESEV PBM antagonizes the ability of NS1 to inhibit activation of the IFN-β promoter by poly(I:C) and RIG-I.

### ESEV PBM impairs NS1 inhibition of IRF3 phosphorylation

**Figure 7:**
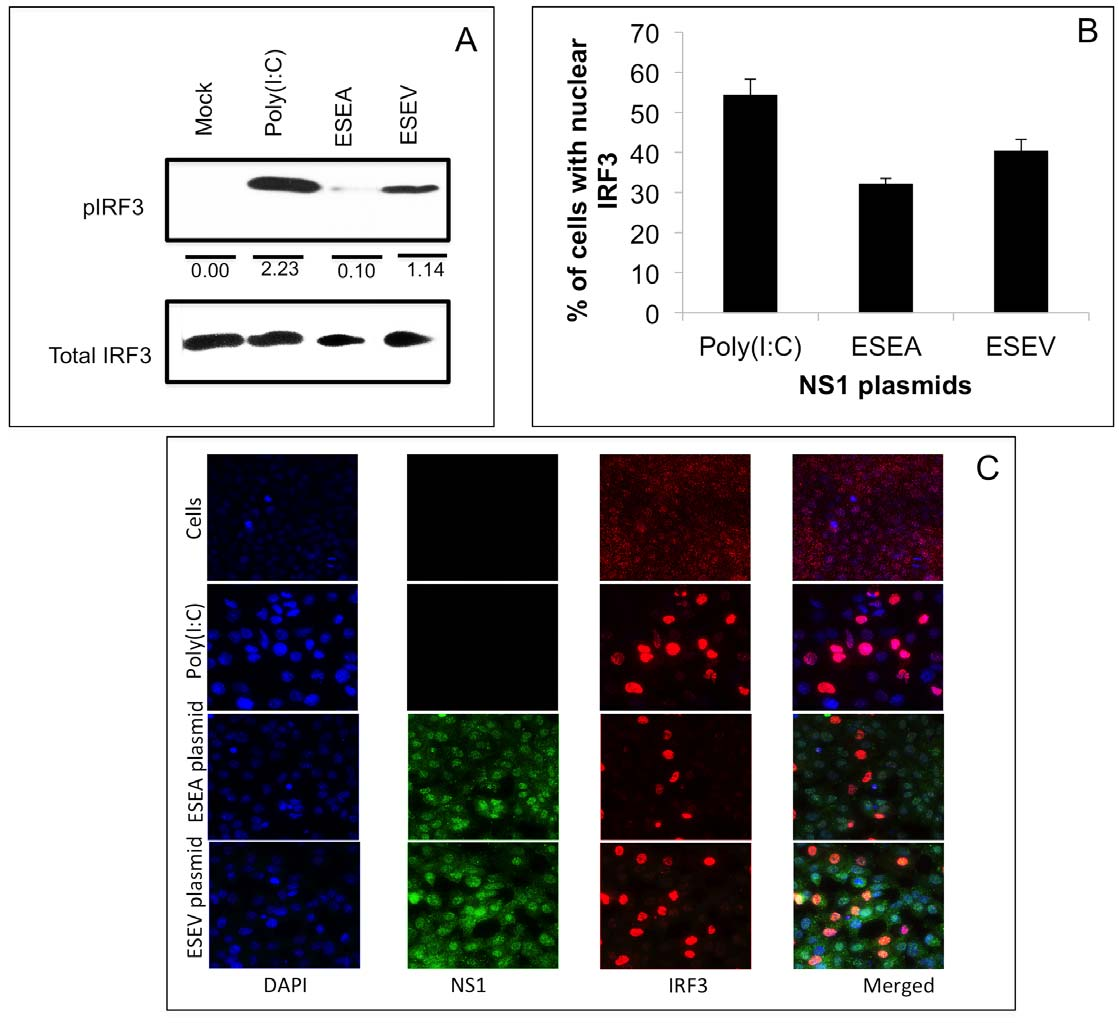
ESEV PBM impairs NS1 inhibition of phosphorylation and nuclear localization of IRF3. A). A549 cells were transfected with wt (ESEV) or PBM mutant (ESEA) NS1 expression plasmids. Cells were re-transfected with poly(I:C) 24 hours later, cell extracts were prepared after 24 hours and levels of phosphorylated IRF3 were evaluated in an immunoblot. Mock-transfected cells were also analyzed. Densitometry was performed with ImageJ software. Values shown are normalized to corresponding total IRF3 levels. B) Percentages of cells with nuclear localized-IRF3 were quantified in three independent experiments by manually counting as described in Materials and Methods and panel C below. A minimum 200 cells were examined in each experiment to quantify IRF3 nuclear localization. Error bars represent the standard error of the mean. Statistical difference in effects of NS1 plasmids was determined by student *t-test* in all the experiments. C). A549 cells were transfected with a wt or mutant ESEA NS1 expression plasmid (with intact CPSF30 binding site). After 24 hours of transfection, cells were activated by transfection of poly(I:C). After 20 hours of activation, cells were processed for immunofluorescence. Nuclei were stained with DAPI; IRF3 is shown as red, and NS1 is shown as green. A representative immunofluorescence images is shown from three independent experiments.

We evaluated the effect of the ESEV PBM on IRF3 phosphorylation following poly(I:C) treatment of A549 cells. We used NS1 expression plasmids with an intact CPSF30 site for these experiments. Cells were transfected with wt or mutant ESEA PBM NS1 expression plasmids and activated with poly(I:C) 24 hours later. After 20 hours of poly(I:C) treatment, levels of phospho-IRF3 (pIRF3) were measured in an immunoblot (Fig. 7A). In the absence of NS1 expression, poly(I:C) treatment resulted in a large increase in the level of pIRF3 as expected. NS1 with the mutant ESEA PBM reduced the amount of pIRF3 in poly(I:C) treated cells >90% relative to that seen with poly(I:C), while expression of NS1 with the wt ESEV PBM reduced phosphorylation of IRF3 ∼50% that seen with poly(I:C) (Fig. 7A). These data further indicate that the ESEV PBM impairs the ability of NS1 to antagonize the IFN response, in agreement with Figures 5 and 6.

As an additional method to examine IRF3 activation, we used immunofluorescence to quantify translocation of IRF3 from the cytoplasm to the nucleus. A549 cells were transfected with wt or ESEA NS1 expression plasmids, 24 hours later cells were re-transfected with poly(I:C), and cells were processed 20 hours later for indirect immunofluorescence to visualize IRF3 and NS1 (Fig. 7C). Although both the wt and ESEA mutant NS1 reduced the percentage of nuclear localized IRF3, the ESEA protein was significantly (p<0.01) more inhibitory than the wt protein (Fig. 7B). These data agree with the pIRF3 immunoblots shown in Figure 7A and further indicate that when expressed from a plasmid, the wt ESEV PBM impairs the ability of NS1 to antagonize the IFN-β response.

### Effect of ESEV on activation of IRF3 and IFN-β promoter during viral infection

**Figure 8:**
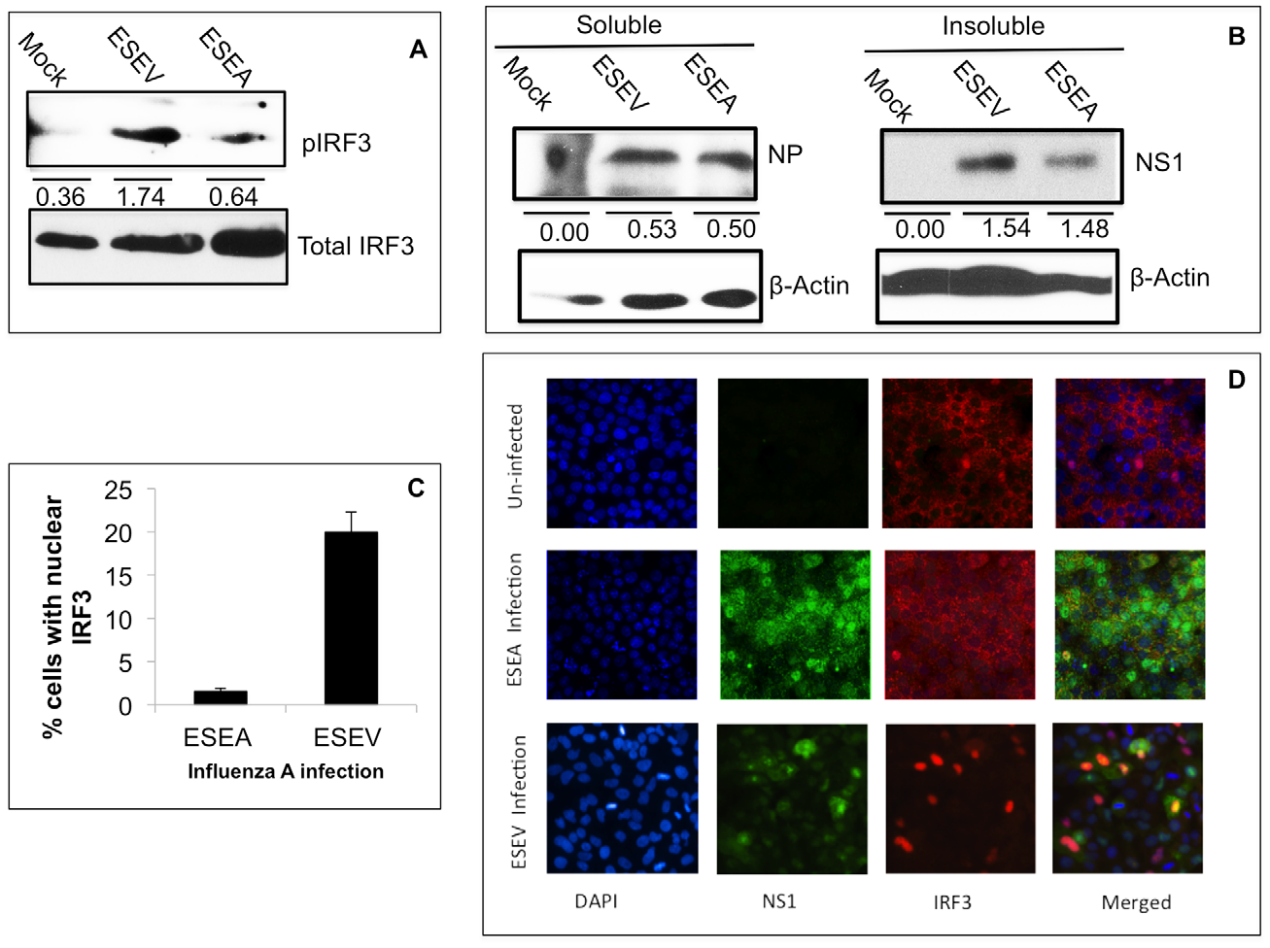
ESEV PBM impairs activation of IRF3 during infection. A). A549 cells were infected at an m.o.i. of 1 with an H3N2 influenza A virus that expresses either an H6N6 NS1protein with the wt ESEV PBM or mutant ESEA PBM virus. Cell lysates were prepared at the 6 hours post-infection and phosphorylated and total IRF3 levels were quantified in an immunoblot. Densitometry was performed with ImageJ software and the pIRF3 levels were normalized to total IRF3. B. A549 cells were infected with indicated viruses and at 6 hours post-infection cell lysates were prepared and separated into soluble and insoluble fractions as described in Materials and Methods; levels of viral NP and NS1 proteins in cell fractions were quantified with ImageJ software and normalized to β-actin levels in the corresponding sample. C) A549 cells were infected an m.o.i. of 1 with an H3N2 influenza A virus that expresses either an H6N6 NS1protein with the wt ESEV PBM or mutant ESEA PBM virus. Percentages of cells with nuclear localized-IRF3 were quantified by manually counting as described in Materials and Methods for three independent experiments. A minimum 200 cells were examined to quantify IRF3 nuclear localization. Error bars represent the standard error of the mean. Statistical difference in effects of NS1 plasmids was determined by student t-test in all the experiments. D). A representative immunofluorescence images used for quantitation in panel C is shown.

We next evaluated the effects of the NS1 ESEV PBM on antagonism of the IFN-β response during viral infection. We used a recombinant H3N2 influenza A viruses (Udorn strain) encoding an H6N6 NS1 protein with either the wt ESEV PBM or mutant ESEA PBM [11], [12]. We examined IRF3 activation in A549 cells infected with either wt virus or a virus encoding a mutant ESEA PBM at six hours post-infection (Fig. 8A). Consistent with the plasmid transfection experiment shown in Figure 7A, pIRF3 levels were ∼2.5-fold higher in cells infected with the ESEV PBM virus than the mutant ESEA PBM virus. The expression of the viral NS1 and NP proteins were monitored at 6 hours post-infection and no significant difference was observed in the levels of the two viral proteins between the wt ESEV PBM virus and the mutant ESEA virus (Fig. 8B). We have previously shown the equal expression of the ESEV and ESEA NS1 up to 8 hours post-infection in a previous study [12]. We also used immunofluorescence to quantify nuclear translocation of IRF3 at six hours post-infection (Fig. 8C,8D). Similar to the immunoblot analysis, infection by the virus that expresses the wt ESEV protein resulted in an increase in nuclear localized IRF3 relative to that seen with infection by the ESEA virus.

**Figure 9:**
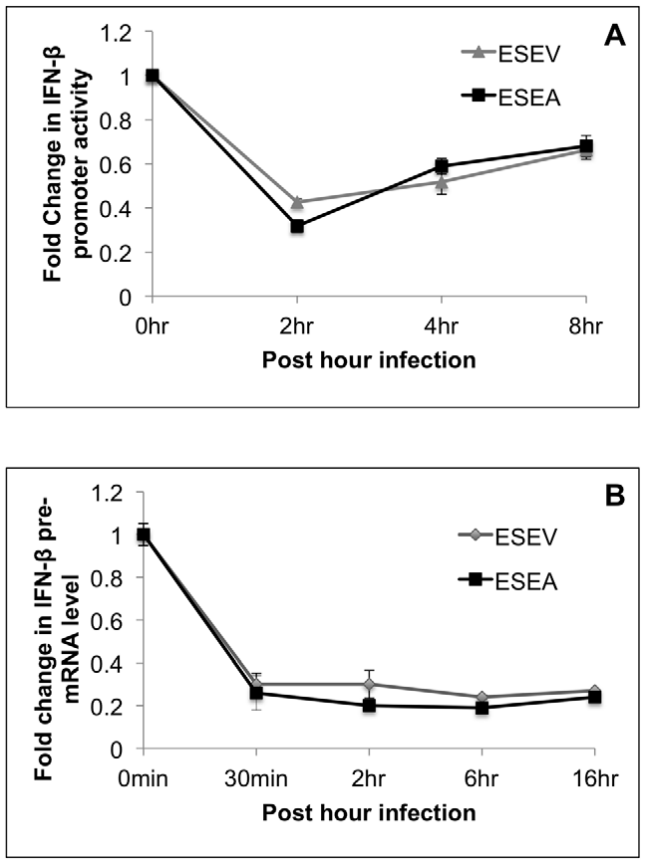
Effects of NS1 ESEA PBM on activation of IRF3 and IFN-β pre-mRNA levels during influenza A infection. A) A549 cells were transfected with IFN-β Luciferase and Renilla Luciferase plasmids. After 24 hours, cells were infected with wt or ESEA virus at an m.o.i. of 1.0. Samples were collected at the indicated times for Luciferase assays. IFN-β luciferase values were normalized to Renilla Luciferase. Error bars represent the standard error of the mean. B) A549 cells were infected with either wt or ESEA virus at an m.o.i. of 1.0. Total RNA was isolated at the indicated times post-infection and RT-PCR assays were performed for IFN-β pre-mRNA as described in Methods. IFN-β pre-mRNA levels were normalized to GAPDH mRNA levels. Error bars represent the standard error of the mean.

Although infection with a virus that expresses an NS1 protein with the ESEV PBM activates IRF3 (Fig. 8A, 8C), induction of IRF3-regulated genes such as IFN-β is unlikely to occur due to viral functions that antagonize the IFN-β response downstream of IRF3 activation, especially the inhibition of IFN-β mRNA 3′ end processing by the interaction between NS1 and CPSF30. To examine induction of the IFN-β promoter occurs during infection, A549 cells were transfected with the IFN-β Luciferase and Renilla Luciferase plasmids and 24 hours later cells were infected with either the wt or ESEA virus. IFN-β promoter activity was measured by Luciferase assays at different time points post-infection (Fig. 9A). IFN-β promoter activity was repressed to nearly identical levels during infections of the wt or mutant ESEA virus. Next, we examine the effect of PBM on IFN-β pre-mRNA level using the RT-PCR assay developed by the Krug laboratory [36]. A549 cells were infected with the wt or mutant NS1 virus and total cellular RNA was isolated at several time points post-infection (Figure 9B). Results of RT-PCR assayed demonstrated that in the context of infection, the wt and ESEA mutant NS1 proteins demonstrated a similar inhibitory effect on IFN-β pre-mRNA levels. These data in infected cells contrast with those in NS1 plasmid transfection experiments (Figs. 5,6) in which the wt PBM reduced the ability of NS1 to antagonize the IFN response. These observations in infected cells indicate that influenza A virus possess multiple mechanisms to counter IFN-β induction such as the viral PB2 and PB1-F2 proteins which inhibit signaling pathways involved in IFN induction [31], [37]. Additionally, the inhibition of CPSF30 by NS1 is likely to dominate over the ESEV PBM antagonism of inhibition of IFN induction.

## Discussion

In this study of PDZ targets of the NS1 ESEV PBM, we found that siRNA depletion of MAGI-I increases the phosphorylation and nuclear translocation of IRF3 and activates the IFN-β promoter (Figs. 1, 2). We also observed that expression of the ESEV NS1 protein during infection perturbs the plasma membrane localization of MAGI-1 (Fig.4). These data suggest that perturbation of MAGI-1 by the ESEV NS1 protein may be sensed in infected cells as a danger signal and trigger the induction of IFN-β. To our knowledge, this is the first report that MAGI-1 is involved in a pathway of IFN induction. In the context of influenza A virus infection, IFN-β is not induced by the perturbation of MAGI-1 by NS1, as the virus encodes multiple anti-IFN functions, including the ability of NS1 to inactivate CPSF30 and prevent maturation of IFN-β mRNA [28]. The viral PB2 and PB1-F2 proteins also possess anti-IFN induction functions [30], [31].

MAGI-1 and the related MAGI-2 and MAGI-3 proteins are members of the membrane-associated guanylate kinase (MAGUK) family. The MAGI proteins contain three protein-protein interaction domains: PDZ, WW, and enzymatically inactive guanylate-kinase-like domains [38]. MAGI-1 is a scaffold proteins involved in the assembly of multiprotein complexes on the inner surface of the plasma membrane and it participates in tight junction formation [39]. MAGI-1 has been shown to interact with a number of cellular protein, including β-catenin, TRIP6, and Slo1 [40], [41]. The interaction between MAGI-1 and β-catenin suppresses Wnt signaling and thereby demonstrates a tumor suppressor function [42]. MAGI-1 also regulates Ca^++^ signaling via its interaction with Slo-1 channel proteins [40] and its activation of Rap1 at sites of cell-cell contract [43]. MAGI-1 clearly regulates processes important for viral infection, as in addition to being targeted by the NS1 ESEV PBM, MAGI-1 is targeted by the PBMs of the adenovirus E4-ORF1 and HPV E6 proteins. Similar to NS1, the E4-ORF1 interaction with MAGI-1 results in sequestration of the cellular protein into cytoplasmic puncta [44]. In contrast, interaction of the E6 protein with MAGI-1 results in the proteasome-mediated degradation of the cellular protein [44], [45].

Our observation that the interaction between the ESEV NS1 protein and MAGI-1 likely activates an IFN-β induction signal was unexpected, as no previous studies have linked MAGI-1 to the innate immune system. However, MAGUK or MAGI-1-like scaffold proteins involved in multiprotein complexes can regulate innate immunity, such as the family of Caspase recruitment domain (CARD)-containing scaffold proteins, known as CARMA. CARMA family members are involved in the signaling cascade induced by activation of cellular receptors such as the T cell receptor that lead to NF-κB activation [46]. The signaling pathway whereby perturbation of MAGI-1 activates IFN-β remains to be elucidated.

The perturbation of MAGI-1 and induction of IFN-β by the NS1 ESEV PBM would seem to be detrimental to influenza A virus replication. However, the ESEV NS1 interaction with Scribble and Dlg1 benefits viral replication – the interaction with Scribble protects infected cells from apoptosis [12], and the interaction with both Scribble and Dlg1 disrupts tight junctions in infected cells and may thereby enhance viral dissemination and spread to new hosts [11]. The specificity of interaction between NS1 and its PDZ targets is conferred almost entirely by only the four residues of the ESEV PBM [11]. With such a limited specificity determinant, NS1 may make interact with some PDZ proteins that confer no benefit to the viral life cycle, and in the case of MAGI-1, may actually be detrimental. However, the benefit derived from targeting such key PDZ proteins as Scribble and Dlg1 may dominate over the negative consequence of binding to MAGI-1. Moreover, influenza A viruses encodes at least three proteins – NS1, PB2, and PB1-F2 –that block IFN induction at multiple points in the induction pathway. Our results highlight the benefit to influenza A virus of encoding multiple proteins that block multiple pathways of IFN induction. It is possible that other viruses that encode PBMs derive similar benefits from encoding multiple anti-IFN induction proteins.

In conclusion, we have found that MAGI-1 is involved in the IFN-β activation pathway. To our knowledge, this is the second PDZ protein found to regulate the IFN system, as Scribble has been shown to be involved in INF-stimulated JAK-STAT signaling. The binding of the Tick-borne encephalitis virus NS5 protein via an internal PBM to Scribble inhibits this signaling [32]. The mechanisms whereby MAGI-I and Scribble regulate the IFN system remain to be identified. Finally, our finding that the NS1 ESEV PBM can target some PDZ proteins that may be detrimental to influenza A virus replication, as well as other PDZ protein that are beneficial to the virus, may provide an explanation for the relative instability of the avian ESEV and human RSKV PBMs between viral isolates. Some of the early isolates of the highly pathogenic H5N1 viruses contain a nonfunctional PBM with an EPEV sequence [11], and the recent swine-origin H1N1 pandemic virus contains a deletion of the PBM. It is possible that loss of a functional PBM is not strongly selected against due to its detrimental PDZ targets, although retention or re-acquisition of a functional PBM remains under some selection pressure due to the beneficial PDZ targets.

## Materials and Methods

### Cells, plasmids and influenza A virus

A549 cells [47] were cultured in Dulbecco's modified Eagle medium (DMEM) with 10% fetal bovine serum (Invitrogen) at 37 degrees C with 5% CO_2_. The plasmids containing the NS1 cDNA (ESEV as PBM) from an H6N6 avian influenza A virus isolate (A/Blue-winged teal/MN/993/1980) was kindly provided by Clayton Naeve (St. Jude Children's Research Hospital). The PBM ESEV was altered to ESEA with a QuikChange site-directed mutagenesis kit (Stratagene). Where indicated, the CPSF30 binding site in NS1 was altered from GLEWN to RFLRY to allow increased expression levels of NS1 proteins from plasmid expression vectors. The MAGI-1 plasmid GW1/HA-MAGI-1b was a kind gift from Dr. Ronald Javier (Baylor College of Medicine). The plasmids encoding full length RIG-I (pEF-BOS-FLAG-RIG-I) was kindly provided by Dr. Takashi Fujita (Kyoto University, Japan) Recombinant viruses that encodes H6N6 NS segment (A/blue-winged teal/MN/993/1980) in an Udorn (A/Udorn/72) background were used for all infections [12]. These viruses expressed H6N6 NS1 with a wild-type ESEV or mutant ESEA PBM. The identities of viral stocks were confirmed by DNA sequencing.

### Plasmid transfections and Luciferase assays

The Interferon-β firefly Luciferase reporter plasmid was kindly provided by Dr. Betty Slagle (Baylor College of Medicine, USA). Luciferase reporter plasmids (IFN-β promoter reporter and Renilla Luciferase reporter) were transfected into duplicate wells (24-well format) of cell cultures using Lipofectamine LTX reagent according to the manufacturer's protocol (Invitrogen). The IFN-β promoter reporter plasmid was activated by dsRNA transfection of 4 µg/ml Poly(I:C) (InvivoGen) at 24 hours post-transfection of Luciferase reporter plasmids. Cells were collected 20 hours after poly(I:C) transfections and processed using a dual-Luciferase assay according to the manufacturer's protocol (Promega Corporation). Mean values of Renilla-normalized firefly Luciferase expression were determined from triplicate readings from duplicate wells. To test the effect of NS1 plasmids with wild-type PBM ESEV or mutant ESEA PBM on IFN-β activation, Luciferase plasmids were co-transfected with either empty vector FLAG-CMV or NS1 plasmids followed by re-transfection 24 hours later with 4 µg/ml poly(I:C); total amount of plasmid DNA transfected per well was held constant at 160 ng. To activate with RIG-I, cells were transfected with IFN-β promoter reporter, Renilla Luciferase reporter plasmid with full length RIG-I plasmid for 24 hr; Luciferase expression was measured as described above and normalized to Renilla Luciferase. At least three independent transfections in A549 cells were performed for each experiment.

### Cell extracts, immunoprecipitations and immunoblots

Cell extracts used for immunoprecipitations were lysed by EBC buffer (50 mM Tris-HCl [pH 8.0], 120 mM NaCl, 0.5% Nonidet P-40, 5 mM dithiothreitol [DTT]) containing protease inhibitor cocktail. To partition cell lysates into soluble and insoluble fractions, cell extracts were spun at full speed in a microfuge, the supernatant was removed and the pelleted material was resuspended in 200 µl loading buffer for SDS-gels. Equal volumes of soluble cell extracts and resuspended pelleted material, representing equivalent numbers of cells, were loaded on SDS-polyacrylamide gels. MAGI-1 was immunoprecipitated using rabbit anti- HA antibody (Sigma) followed by addition of protein A bead to precipitate the immune complex. NS1 was immunoprecipitated using anti-FLAG M2 beads (Sigma). Products of immunoprecipitations were run on SDS-polyacrylamide gels and were transferred to nitrocellulose membranes and probed with appropriate antiserum. Antisera used for immunoblots were anti-MAGI-1 (Sigma at 1∶1000 dilution), anti-NS1 (Immune Technology at 1∶1000 dilution), anti-Dlg1 (SAP97 from Santa Cruz Biotechnology at 1∶500 dilution), anti-HA (Sigma, at 1∶1000), anti-FLAG (Sigma, at 1∶1000), Scribble (Santa Cruz used at 1∶500), total IRF3 (Abcam, at 1∶500), β-actin (Abcam, at 1∶10000) and pIRF3 (Cell Signaling at 1∶1000).

### Immunofluorescence

Immunofluorescence was performed as described previously [11], [12]. A Z series of focal planes was captured and deconvolved using the Applied Precision DeltaVision restoration microscopy system with the softWoRx software program (Applied Precision). A single focal plane from each Z series was then further processed in Adobe Photoshop. To quantify IRF3 nuclear localization, A549 cells were stained with anti IRF3 (Abcam at 1∶20 dilution) and anti-NS1 antiserum (used at 1∶100 dilution). The IRF3 nuclear localization was examined in at least 200 cells from three independent experiments. To determine the co-localization of MAGI-1 and NS1, antisera for MAGI-1 (Sigma used at 1∶200), NS-1 (Immune technology at 1∶100) and Scribble (Santa Cruz used at 1∶50) were used.

### Real Time PCR for IFN-β mRNA

Total RNA from infected or transfected A549 cells was extracted using RNeasy Kit (Qiagen) according to the manufacturer's protocol. Quantitation of IFN-β pre-mRNA was performed according to protocol described by Kuo et al 2010 [36]. The amount of pre-mRNA and mature mRNA was determined by calculating Ct values using Bio-Rad multicolor Real-Time detection system (Bio-RAD). The fold-changes in IFN-β pre-mRNA level were calculated by a deltadelta CT method [48].

### siRNA depletions

SiRNA against MAGI-1 was purchased from Sigma Aldrich; siRNAs against Dlg1 and Scribble were purchased from Santa Cruz Biotechnology. SiRNAs (10 ng/well for Dlg1, Scribble, control siRNA and 18 ng for MAGI-1) were transfected into A549 cells (24 well cultures dishes) using RNAimax according to the manufacturer's reverse-transfection protocol. Data was analyzed from three reading from duplicate wells. The IFN-β promoter activity, IFN-β pre-mRNA level, IRF3 phosphorylation, and IRF3 nuclear localization in siRNA-depleted cells were measured as described above.
